# Ligand- and Structure-based Approaches for Transmembrane Transporter Modeling

**DOI:** 10.2174/2589977515666230508123041

**Published:** 2023-05-24

**Authors:** Melanie Grandits, Gerhard F. Ecker

**Affiliations:** 1 Department of Pharmaceutical Sciences, University of Vienna, Vienna, Austria

**Keywords:** ABC transporter(s), breast cancer resistance protein (BCRP), *in silico* modeling, machine learning, P-glycoprotein (P-gp), quantitative structure-activity relationship(s) (QSAR), solute carrier (SLC) transporter(s)

## Abstract

The study of transporter proteins is key to understanding the mechanism behind multi-drug resistance and drug-drug interactions causing severe side effects. While ATP-binding transporters are well-studied, solute carriers illustrate an understudied family with a high number of orphan proteins. To study these transporters, *in silico* methods can be used to shed light on the basic molecular machinery by studying protein-ligand interactions. Nowadays, computational methods are an integral part of the drug discovery and development process. In this short review, computational approaches, such as machine learning, are discussed, which try to tackle interactions between transport proteins and certain compounds to locate target proteins. Furthermore, a few cases of selected members of the ATP binding transporter and solute carrier family are covered, which are of high interest in clinical drug interaction studies, especially for regulatory agencies. The strengths and limitations of ligand-based and structure-based methods are discussed to highlight their applicability for different studies. Furthermore, the combination of multiple approaches can improve the information obtained to find crucial amino acids that explain important interactions of protein-ligand complexes in more detail. This allows the design of drug candidates with increased activity towards a target protein, which further helps to support future synthetic efforts.

## 1 INTRODUCTION

Cells and their internal compartments are protected by phospholipid bilayers, which are of fundamental importance for the separation of internal and external milieus. Being hydrophobic by nature, the lipid barrier limits the movement of mostly hydrophilic solutes, such as metabolites, neurotransmitters, hormones, drugs, and xenobiotics. However, it does not provide a completely impermeable barrier, as cells have evolved mechanisms to, on one hand, selectively accumulate individual compounds, and, on the other hand, export xenobiotic or toxic compounds. This is achieved by the large and heterogenous family of transmembrane transport proteins, which comprises in humans more than 600 genes, clustered into two superfamilies: the ATP binding cassette (ABC) [1, 2] transporters and the solute carriers (SLCs) [3, 4]. While ABC transporters are generally involved with the (unselective) export of drugs, SLCs have been mostly described to be involved in (selective) compound uptake. However, a subset of SLCs has been linked to an export function, such as MATE (multidrug and toxic compound extrusion) transporters [5].

Based on their instrumental role in drug absorption, distribution, and excretion, considerable knowledge has accumulated on several ABC-transporters (multidrug resistance protein 1 (MDR1), breast cancer resistance protein (BCRP), multidrug resistance-associated protein 1 (MRP1)) [6], as well as the large SLC22 and SLCO subfamilies of SLCs [7, 8]. The important role of these transporters in governing the pharmacokinetics of several drugs has also been corroborated by several pharmacogenomic polymorphisms [9]. Accordingly, the US Food and Drug Administration and the European Medicines Agency recommend testing several of these transporters for clinical drug interaction studies (Table **1**) [10].

While for most of the ABC-transporter, their physiological role is known. SLCs represent a largely understudied family, counting more than 460 members of which only a very modest fraction is targeted by drugs. In fact, 40% of all SLCs must be considered orphan, because neither their substrate nor any inhibitor is known. To overcome this huge knowledge gap, RESOLUTE (Research Empowerment on Solute Carriers), a project under the framework of the Innovative Medicines Initiative (IMI), aims at deorphanizing at least 72 SLCs. The RESOLUTE project is a public-private partnership with 13 partners from academia and industry with the goal to establish SLCs as a tractable target class for medical research and development. With a systematic and coordinated effort, the project will advance the knowledge of SLC transporter in general and build the SLC knowledgebase by combining public and consortium-derived data [11].

In the past decades, computational methods have become an integral toolbox in the drug discovery and development process. Starting with simple regression models based on physicochemical descriptors (Quantitative structure-activity relationship - QSAR), ligand-based modelling advanced to 3D-QSAR and pharmacophore-based approaches. When the first protein structures became available, structure-based techniques were employed. Furthermore, with the increasing availability of large public data sources, such as ChEMBL [12], PubChem [13], Transporter Classification Database (TCDB) [14], Metabolism and Transport Database (Metrabase) [15], and integrated versions like the Open Pharmacological Concepts Triple Store (Open PHACTS) [16], machine learning (ML) and even deep learning were pursued. In this short review, a set of cases on computational approaches for the prediction of ligand-transporter interactions is provided, focusing on selected ABC, as well as SLC-transporters. Fig. (**1**) represents the methods discussed in this review and provides an overview of the association between them. While QSAR and pharmacophore modeling is often used in a ligand-based approach, docking and molecular dynamics simulations use the protein structure as a starting point. Combining the output of these methods offers more insight into the protein-ligand complex. Furthermore, more advanced methods, like machine learning, can use this additional information as input to generate prediction models. Also, the outcome of machine learning approaches can be used to improve ligand- and structure-based approaches.

## 2 LIGAND-BASED MODELING APPROACHES

### 2.1 QSAR-studies of P-glycoprotein (P-gp) Inhibitors

Regarding ABC-transporter, a lot of effort has been put towards the design and synthesis of inhibitors due to the involvement of some of these proteins in cancer multidrug resistance [17]. These are P-glycoprotein (P-gp), the breast cancer resistance protein (BCRP), and the multidrug resistance-associated protein 1 (MRP1). The challenge for all these proteins is their polyspecificity. For P-gp, the paradigm protein for this family, a large number of chemical scaffolds for inhibitors have been published, with an overall commonality of high lipophilicity, a basic nitrogen atom (which has been questioned later on), and at least one aromatic ring [18]. Interestingly, within all series, conclusive structure-activity relationships could be derived, which translates to clear determinants for high and low inhibitory activity. Examples are manifold and comprised of, *e.g*. QSAR studies on propafenone analogs, steroids (Example: Fig. (**2**), [19]), flavonoids, and many other chemical scaffolds [20]. These models usually consist of extracting molecular descriptors from the compounds of interest (2D descriptors for Hansch analysis, or 3D descriptors for 3D-QSAR) and then combining them *via* multiple linear regression analysis to predict a continuous endpoint like IC_50_ values. In all these cases, the computational models focused on narrow chemical compound classes, which limits general applicability. Thus, these models are not suited for predictions beyond the narrow chemical space of the training set. Therefore, approaches to building consensus models were employed by integrating local models from multiple chemical series [21]. One of the most systematically analysed scaffolds is propafenone, a sodium channel blocker marketed as an anti-arrhythmic drug. Systematic variations showed that most of the changes affect P-gp inhibitory activity mainly *via* their contribution to the overall lipophilicity of the molecules. Even when pharmacophoric features, such as the carbonyl oxygen, are modified, *i.e*. by transforming the basic phenone scaffold to a benzofurane, this behaviour stays [22].

**Fig. (3):**
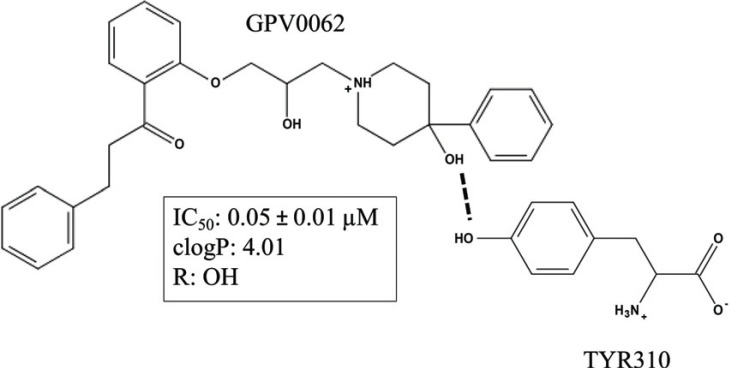
Hydrogen bonding and lipophilicity as key contributors to inhibitory activity towards P-gp. A study by Cseke *et al*. [24] reported that not only a high logP of a compound is essential for an inhibitory activity but also hydrophilic interactions of the piperidine ring. Compound GPV0062 was highlighted as a representative of a propafenone analog with a high inhibition capacity, providing an additional interaction due to its 4-hydroxy group. The relevant interaction between the compound and Tyrosine 310 is presented with dashed lines. The IC_50_ values are obtained from three independent experiments and the values are the mean ± standard deviation. clogP is the calculated partition coefficient of octanol/water and was determined using the MarvingSketch tool from ChemAxon. R is the substituent at the piperidine ring.

However, modifications in the vicinity of the nitrogen atom indicated that hydrogen bonding plays an important role. This led to the discovery of GPV0062, a hydroxyphenypiperidine analog of propafenone, which shows one order of magnitude higher activity than predicted solely on the basis of its lipophilicity. Moreover, also for this series, there is a basic underlying correlation with the overall lipophilicity of the compounds [23]. The study by Cseke *et al*. [24] demonstrated that an increase in activity/logP ratio is due to hydrogen bonding acceptor features of the 4-hydroxy group at the piperidine ring (Fig. **3**). This was also supported by a structure-based approach. Furthermore, the vicinity of the nitrogen atom is generally crucial, as also basicity/hydrogen bond acceptor features of the nitrogen, as well as the size of the substituent, play a key role [25]. Last but not least, implementing the Topliss approach pointed towards the importance of electronic contributions of substituents at the two aromatic rings. All these systematic Hansch-type QSAR studies finally led to the design of GPV0576, exhibiting P-gp inhibitory activity 1000-fold higher than the lead compound propafenone. Analogous studies have been reported for other scaffolds, such as tariquidar [26] and phenothiazines [27]. This type of regression model has proven useful for a deep understanding of a certain chemical compound class with respect to its interaction with a certain transporter, directing toward future synthetic efforts.

### 2.2 Ligand-based Pharmacophore Modeling

**Fig. (4):**
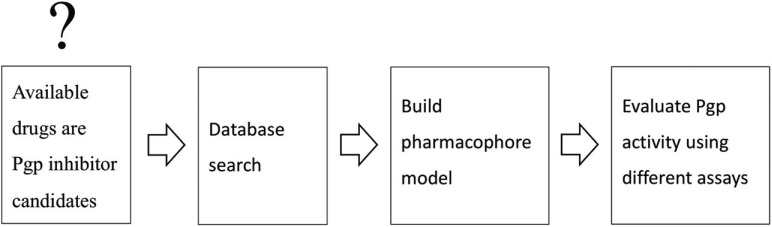
Simplified representation of a pharmacophore-based virtual screening study. The necessary steps, from formulating a hypothesis to further testing it, with an *in silico* approach as well as experimental studies. As an example, P-gp inhibitors from known drugs were detected using pharmacophore-based virtual screening and various assays as a validation step [28].

Pharmacophore modelling is an excellent tool to derive basic pharmacophoric features across different chemical scaffolds. Moreover, in the case of ABC-transporters, numerous pharmacophore models have been derived, which show good capabilities in identifying new ligands with new chemical scaffolds, thus proving their utility. These comprise pharmacophore models for flavonoid-type P-gp inhibitors (Fig. **4**) [28], as well as for ligands of BCRP [29] and the bile salt export pump (BSEP) [30]. In all cases, experimental validation of the models was pursued by using the pharmacophore model for screening a compound library and experimental testing of the top-ranked hits. Also, for propafenone-type inhibitors, pharmacophore models were developed and allowed to identify new P-gp inhibitors [31]. For this scaffold, also a selectivity profiling approach for P-gp/BCRP was pursued, which allowed identifying pharmacophoric features favouring one or the other transporter [32]. This profiling was complemented by a study that aimed at morphing the P-gp selective propafenone scaffold gradually towards the BCRP selective fumitremorgin scaffold [33].

### 2.3 Machine Learning Approaches

With the availability of large, public data sources, the focus shifted to classification models. One of the ground-breaking contributions is the work of Broccatelli *et al*. [34], who combined molecular field analysis, pharmacophore-based descriptors, as well as physicochemical parameters to develop both global and local models for P-gp inhibitors. Based on a data set of 1275 compounds derived from 61 references, the authors identified flexibility, hydrophobic surface area, and logP as the main discriminating physicochemical parameters for inhibitors/non-inhibitors of P-gp. Furthermore, shape also emerged as a crucial factor, indicating the importance of the 3D description of the molecules. Furthermore, the study by Chen *et al*. highlighted the importance of fingerprints in classification models. Fingerprints are used to represent molecular features in a quantitative way. The introduction of fingerprints remarkably improved the prediction accuracy of the models and furthermore allowed to identify the molecular fragments, which are favourable or unfavourable for P-gp inhibition [35].

For BCRP, Montanari *et al*. [36] built a Bayesian classification model using an extended connectivity fingerprint (ECFP) 6. This allowed to extract important substructures, which are mostly in line with currently published SAR studies on BCRP inhibition. For BSEP, Warner *et al*. [37] used data from a membrane vesicle-based BSEP inhibition assay to quantify transporter inhibition for a set of 624 compounds to develop a classification model using a support vector machine (SVM) classifier with a high prediction power, showing an accuracy of 0.87. Also, for this transporter, lipophilicity and molecular size are significantly correlated with inhibition.

Further models for hepatic steatosis were built based on an *in vivo* data set measured in rodent studies. The main issue, in this case, was the imbalance (1:8, minority class: steatotic compounds) of the data, which was overcome by using meta-classifiers-bagging and under-sampling. By comparing different descriptor combinations, the results revealed that the best-performing model (balanced accuracy: 0.7, the calculation of the balanced accuracy in case of imbalanced datasets was more appropriate than the accuracy) was trained with physicochemical features as well as ToxPrint features. ToxPrint is a reference set of structural features that encode physicochemical, atomic, and electronic properties in addition to substructural connectivity. The study also strengthens the advantage of using conformal predictions [38].

Concerning SLC transporters linked to absorption, distribution, metabolism, excretion, and toxicity (ADMET), a set of consensus classification models were built. Kotsampasakou *et al*. [39] built such models for organic anion transporting polypeptide 1B1(OATP1B1) and organic anion transporting polypeptide 1B3 (OATP1B3). These inhibition models are based on 1700 compounds extracted from the literature. This study was extended to organic anion-transporting polypeptide 2B1 (OATP2B1) and allowed to identify specific scaffolds prone to selectivity towards one or the other transporters [40].

SLC transporters are an attractive drug target class [11] given that these transporters play a role in cellular metabolism and disease management [4]. Several studies highlighted that there are many orphan targets in this protein family. The study by Meixner *et al*. helped to identify potential substrates for several orphan SLCs using a machine-learning approach based on a substrate-based ontology [41]. Furthermore, Burggraaff *et al*. used proteochemometric modeling to discover novel compounds for inhibitors of the sodium-dependent glucose co-transporter 1. This approach was used to enhance the chemical space of the model without knowing the structure of the target [42].

In order to provide such classification models to the scientific community, Montanari *et al*. built a web service (https://livertox.univie.ac.at) that allows the prediction of substrates, inhibitors as well as toxic compounds for selected transporter proteins [43]. The available transporters were chosen based on the guidelines from regulatory agencies, which indicate these proteins to be important for routine testing in studies on new drugs. Furthermore, the availability of enough data to build and thoroughly validate these classification models was a criterion. Models predicting the possible transport of compounds were based on a dataset that correlated toxicity with expression levels of several ABC-transporters [44]. The class imbalance present in the substrate datasets was handled by MetaCost [45]. Different machine learning algorithms using publicly available descriptors were tested and the models giving the best cross-validation results (balanced accuracies: 0.64-0.88) were provided to the web service.

Fig. (**5**) shows a general approach on how to build a machine learning model, starting with the identification of an appropriate target, collecting data, and cleaning them as well as choosing the right descriptors and classifier to generate a model with a high predictive power and a broad chemical space. The final validation step is necessary to ensure the applicability of the model.

### 2.4 Structure-based Modeling

With the increasing availability of protein crystal structures both for ABC- and SLC-transporter, structure-based modelling approaches were pursued. However, in the case of ABC-transporter, there are still only very few structures that have been co-crystallised with a small molecule inhibitor. Furthermore, in the case of P-glycoprotein, the most prominent ABC-transporter, there is still no protein structure co-crystallized with a classical substrate/inhibitor, such as verapamil or cyclosporin. In addition, the transporters undergo a substantial conformational change when progressing through the transport cycle, which cannot be covered by a single structure. Furthermore, the huge binding zone with an estimated size 10 times larger than a classical ligand-protein binding site poses an additional layer of complexity. Finally, photoaffinity labelling studies, for instance, showed that there might be simultaneous binding of ligands at different areas of the binding cavity [46]. This requires sophisticated validation protocols for binding hypotheses obtained by ligand docking, which go far beyond, *e.g*. consensus scoring or induced fit docking. In this respect, two main criteria apply:

-) The pose must allow explaining the structure-activity relationship obtained for a series of compounds.

-) The pose must be predictive in either allowing to identify new ligands and/or to predict the effect of a distinct mutation.

In our quest to provide sound binding hypotheses for a selected transporter, we developed an approach named experimental data-guided docking and applied it for docking to P-gp [47], the serotonin transporter (SERT) [48], as well as the benzodiazepine binding site of the gamma-aminobutyric acid (GABA) receptor [49]. The protocol exhaustively samples the pose space of a set of ligands that show a distinct SAR pattern and subsequently clusters the poses according to the common scaffold. Then, the different clusters are analysed with respect to their ability to explain the SAR known for the ligands. In the case of the propafenone analogues, this example implies the importance of the carbonyl group and the 4-hydroxy group at the piperidine ring, as well as the unimportance of the hydroxy group of the propanolamine side chain. The validation of the best pose was performed in two different ways. First, Tyrosine at positions 307 and 310 was mutated to Phenylalanine, anticipating a loss of affinity of the ligand. Strikingly, the mutants showed almost equal activity. Analysing the homology model of P-gp and the binding area in more detail showed that all poses were located at one of the two possible entry gates for the ligands. This is due to the asymmetry of the template structure (mouse P-gp), which renders the space at the second entry gate quitenarrow. At the second gate, which, under physiological conditions, should be equally accessible, there are 6 Tyrosines, which were not mutated and easily might take over (Peter Chiba, unpublished). This points towards the uncertainty of using site-directed mutagenesis for the validation of docking poses, especially when dealing with highly flexible and promiscuous transport proteins. Second, the pose served as a blueprint for a structure-based pharmacophore model, which was subsequently used for the virtual screening of the Enamine library. Top-ranked hits were ordered and experimentally tested. This identified two novel P-gp inhibitors with an activity in the sub-micromolar range (K. Prokes, diploma thesis, Uni. Vienna 2017). The important role of the 4’-hydroxyl group at the pipieridine moiety as a hydrogen bond acceptor was further strengthened by synthesizing and testing a series of compounds with varied substituents in this position [22].

A different approach was used by Ferreira *et al*. [50]. Using a previously refined structure of murine P-gp, they identified the M- (verapamil), H- (Hoechst 33342), and R-site (rhodamine-123) of P-gp by means of molecular docking. The drug-binding pockets were defined as substrate- or modulator-binding sites according to the molecules that preferentially docked in each of the sites. In this annotation, “modulator” refers to compounds that appear to block the efflux of substrates. These substrates are supposed to bind to either the H or the R site. Analogously, the modulator-binding (M) site was linked to the main interaction site for verapamil. In order to validate their protocol, the authors performed further docking studies with molecules classified as substrates or modulators in order to retrieve a structure-based classification model with the ability to discriminate substrates from modulators. The final model properly predicted 14 out of 19 modulators (74%), 20 out of 32 substrates (63%), and 2 out of 3 non-substrates. Although being quite predictive with their model, the authors concluded that the flexibility of the protein should be taken into account, which emphasizes the importance of molecular dynamics simulations for further insights.

Indeed, in a different publication [51], the authors performed a series of 100 ns molecular dynamics simulations in order to refine their homology model. Subsequent production was made with a small set of ligands, providing valuable insights into the molecular interactions established between several ligands and the drug-binding pocket and allowing to distinguish inhibitors from substrates. Thus, the modulators studied consistently established a higher number of nonbonded interactions (1.1 to 2.3), mainly aromatic ones, when compared with substrates.

These two examples convincingly demonstrate that structure-based modeling concerning ABC-transporter has become a valuable tool for a deeper understanding of the molecular features driving ligand–transporter interaction. However, there is still a lack of high-quality structures for most of the ABC-transporter subfamilies. Making protein structures available, such as the one for human BCRP [52], helps to improve structure-based studies for this transporter. Unfortunately, there is currently no structure available for the bile salt export pump BSEP, which is an important target for the prediction of hepatotoxicity.

Regarding the SLC transporter, the structure of the human serotonin transporter simultaneously complexed with 2 inhibitors marks a breakthrough in the field. It outlines for the first time the concrete location of an allosteric binding site at SLC transporters, which opens a whole new field of study [53]. Furthermore, Colas *et al*. proposed the extensive mapping of structure-function relationships of multiple transporters with the same fold to understand especially secondary sites of SLCs [54]. It was also suggested that not only the structure-based information is of interest, but also the sequence information of the protein to get a full picture, which should help to understand why unrelated SLCs can transport the same substrates [55].

### 2.5 Molecular Dynamics (MD) Simulations

As mentioned before, MD simulations are used to study the interactions of compounds and their corresponding target on a molecular level. It provides more insight into these interactions by providing a dynamic picture compared to other methods like molecular docking, where only static information is provided and a certain environment (*e.g*. phospholipid membrane) cannot be taken into account. Although MD simulations provide a deeper understanding of protein-ligand complexes, this method suffers due to time-consuming and resource-intensive tasks as well as parameterization efforts (inadequate parameterization of the compound). Therefore, several studies were undertaken to overcome these obstacles, with an aim to not only improve the parameterization of the compound but also to enhance molecular dynamics algorithms to allow more sampling in a shorter time frame as well as combining MD simulations with other approaches to overcome these issues [56]. Furthermore, the optimization of the algorithms to take full advantage of recent hardware innovations as well as performing the calculations on co-processors, such as graphics processing units (GPUs), could speed up the tasks.

However, not only enhanced sampling methods are of interest, but also methods that allow a more precise free energy calculation are required. The study by Ries *et al*. [57] tried to tackle this issue by creating a Python package called Ensembler that allows method prototyping by using basic techniques as well as enhanced sampling and free energy approaches. It further eases the way of sharing scientific code by making it readily available to other scientists.

Subramanian *et al*. [58] used the enhanced sampling method Umbrella Sampling to find binding locations for competitive and noncompetitive P-gp substrates, providing insight into a general scheme for these interactions.

The simulation of transporters is also very challenging due to the high flexibility of the protein and the lack of high-resolution crystal structures available. Furthermore, the physiologically relevant environment for the protein needs to be considered. In the study by Condic-Jurkic *et al*. [59], different structural models were used for the simulations of P-gp in a membrane. It was concluded that the nucleotide-binding domains, important for ATP binding, changed significantly during the simulation. Compared to the transmembrane domain, important for the binding of compounds, it seems more stable, especially for the model based on the 4M1M (PDB ID) target. It was concluded that the time frame (100 to 200 ns) of the simulations was not long enough to explain the interaction pattern of a flexible protein of this size without knowing the initial structure.

Furthermore, MD simulations can be combined with other approaches like molecular docking. This combination provides multiple binding poses of the compound in the binding site as well as helps to find alternative binding sites (*e.g*. allosteric sites) in the target. However, other methods like machine learning can be used to complement molecular dynamics simulations. Here, the information collected during the simulation is encoded in such a way that it is used as input for the training of the machine learning model [60]. In addition, there are approaches that help to improve the simulations by using machine learning by improving the sampling and accuracy of simulations. ML is used prior to the simulation to optimize the parameterization of the system [61, 62], but also after the simulation to post-process the results [61]. However, it can also be used to enhance the accuracy of the simulations by providing a more advanced set-up as well as improve the analysis of the results [60].

Riniker *et al*. performed short MD simulations in different environments to develop a fingerprint named Molecular Dynamics Fingerprints (MDFP), which can be used for the training of the machine learning models used to predict solvation-free energies [63]. Furthermore, it was reported that this approach helped to find Pgp-substrates by using fingerprint as an orthogonal descriptor for the training of a classification model [64].

Fig. (**6**) gives an overview of how molecular dynamics simulations can be combined with other *in silico* methods like molecular docking and machine learning to support these approaches and *vice versa*.

### 2.6 Combining the Best of Two Worlds

As outlined above, the number of available 3D structures of ABC proteins combined with the performance of experimental approaches laid the ground for the application of structure-based methods to predict drug/transporter interaction. Besides a set of models based on rather small data sets, the study by Klepsch *et al*. reported that structure-based classification for P-gp inhibitors is within reach [65]. It started with Dolghih *et al*., who used induced fit docking into the crystal structure of mouse P-gp to separate P-gp binders from nonbinders on the basis of their docking score [66]. They concluded that by using flexible docking, the Glide XP score was -14 or lower for compounds showing a P-gp interaction, while for compounds showing no interaction, the score was mostly -12 or greater. Furthermore, docking scores with values between -12 and -14 show less conclusive results. Chen *et al*. tried to classify substrates and non-substrates of P-gp based on Glide docking scores; however, no clear separation of the two classes could be observed [67]. Klepsch *et al*. applied supervised machine-learning and structure-based techniques to predict P-gp inhibitors and noninhibitors, using a structurally diverse data set of more than 1000 compounds [47, 65]. The methods applied comprised ligand-based supervised machine learning methods, such as random forest, decision tree, support vector machine, κ-nearest neighbour, and binary QSAR, as well as structure-based docking studies on a homology model of P-gp based on the mouse P-gp structure using five different scoring functions (ChemScore, GoldScore, Astex Statistical Potential (ASP), ChemPLP, and XScore). Although the performance of the structure-based classification was considerably lower (61% for the external test set) than those obtained by Random Forest or SVM classification (73% and 75%, respectively), it showed that structure-based classification of ligands of ABC-transporter was within reach. In addition, this further validates the protocol used for docking small molecules into P-gp outlined above.

An analogous approach was used for BSEP. BSEP actively transports conjugated monovalent bile acids from the hepatocytes into the bile. This facilitates the formation of micelles and promotes digestion and absorption of dietary fat. Thus, inhibition of BSEP leads to the accumulation of cytotoxic bile salts in the liver, which might result in drug-induced cholestasis or liver injury. Jain *et al*. constructed a homology model of BSEP based on the corrected mouse P-glycoprotein structure (PDB ID: 4M1M) and used it for docking-based classification of a set of 1212 compounds (405 BSEP inhibitors, 807 non-inhibitors) [68]. Using the scoring function ChemScore, a prediction accuracy of 81% on the training set and 73% on two external test sets could be obtained. Furthermore, analysis of the protein-ligand interaction fingerprints revealed certain functional group/amino acid residue interactions that could play a key role in ligand binding. Also, in this case, ligand-based models, due to their high speed and accuracy, remain the method of choice for the classification of BSEP inhibitors. However, the structure-assisted docking model demonstrated reasonably good prediction accuracies while additionally providing information about putative protein-ligand interactions.

### 2.7 Data-driven Approaches

Although we currently face a tremendous increase in the availability of sufficiently large data sets for ligand-transporter interactions, building robust ligand-based *in silico* transporter models is still limited to a relatively small set of transporters. Data sources for small compound transporter bioactivity data open to the public include specialized transporter databases like TP-search [69], Metrabase (Metabolism and transport database), and UCSF-FDA TransPortal [70], as well as large compound collections, such as ChEMBL or PubChem. However, concerning the integration of diverging levels of data (gene expression data, pathway data, disease, functional, or phenotypic annotations, *etc*.), there is still a lot of work to do. Although the Open PHACTS project laid the ground for large-scale semantic data integration in the area of life sciences, its sustainability beyond the lifetime of the IMI-funded project proved difficult. Furthermore, one of the specialties to be considered in the case of transporter data is the differentiation of substrate, modulator, and inhibitor. In ChEMBL, for instance, the assay descriptions follow no systematic classification other than the determination of the assay type, assay format, and cell line/tissues used [71]. Thus, it is impossible to identify identical assays just on the basis of this narrative description. In the case of human P-glycoprotein, the effort was taken to manually compare those assays available in ChEMBL [72] in order to compile a data set with validated IC_50_ values for further QSAR studies. However, it finally turned out that there is not enough overlap of compounds measured in different assays to allow transforming data from one assay to another. Thus, the hitherto most promising way is to compile binary sets of data (inhibitor/no inhibitor) and develop classification models. In this case, the definition of the respective threshold is of crucial importance and requires expert knowledge in order to retrieve high-quality data sets. A proper process for this has been demonstrated by Montanari *et al*. [36], who collected data for BCRP inhibition from 47 sources (publications and PubChem bioassays) and applied assay-tailored thresholds to distinguish inhibitors from non-inhibitors. This led to a data set containing a total of 978 compounds, which allowed the development of predictive classification models.

However, the real power of publicly available large data sources can be exploited when extracting data using workflow engines, such as the Konstanz Information Miner (KNIME) [73]. These allow to compile complex queries, such as the one used by Montanari *et al* [74], which collects multi-label data from the Open PHACTS discovery platform. Using inhibition data of P-gp and BCRP, the authors applied the multi-class classification to gain insights into the driving features for the selectivity of inhibitors for one transporter over the other. Aniceto *et al*. used the same methods to predict P-gp, BCRP, MRP1, and multidrug resistance-associated protein 2 (MRP2) substrates with data from Metrabase [75]. A step even further to multilabel classification is proteochemometrics modelling (PCM). PCM combines ligand information as well as target information in order to predict an output variable of interest (*e.g*. activity of a compound). The big advantage of PCM compared to conventional Quantitative Structure-Activity Relationship (QSAR) modeling is that by creating a single model one cannot only predict the affinity of a diverse set of compounds to a diverse set of targets but also extrapolate the specific ligand-protein interactions that might be relevant for binding. Moreover, by combining different datasets with sparse data availability can be included and modelled. PCM has been successfully applied to SLC and ABC transporters [76]. In the work by Kickinger *et al* [77], it was used to predict the activity of neurotransmitter transporter, such as the four GABA transporters (gamma-aminobutyric acid transporter (GAT) 1-3, betaine/gamma-aminobutyric acid transporter (BGT) 1) and could be subsequently used for all transporters of the SLC6 family. As a basis for creating the data set, a respective KNIME workflow was developed. Overall, the authors observed that PCM models showed similar performance as individual QSAR models for each transporter. Remarkably, by analyzing the importance of the descriptors used in the PCM models, residues that have been already confirmed in mutational studies as being relevant for binding, were ranked very high. Thus, PCM is a new approach that not only helps to identify new active molecules on transporters but is also capable of shedding light on the possible binding modes of these compounds.

Another example of retrieving congeneric and consistent SAR data sets for protein targets of interest is the work by Zdrazil *et al*. [78]. Starting from the two phylogenetically related transporters, SERT and dopamine transporter (DAT), the whole chemical compound space for inhibitors was explored by implementing a scaffold-based clustering of compounds possessing biological measurements for both targets. The data retrieved not only allowed to study selectivity trends of scaffold series but also enabled us to derive QSAR equations for a set of 56 cathinone analogues for both SLC transporters. These QSAR equations triggered the refinement of binding hypotheses obtained from docking of the compounds into protein homology models of SERT and DAT. This delivered useful insights into driving determinants for human DAT selectivity over human SERT.

Finally, KNIME workflows can also be used to provide an overview of available data for a whole protein family, such as SLC transporters. Querying a set of public databases for these transporters to retrieve interacting ligands helps to distinguish well-characterized SLCs from the ones with basically no information available. Furthermore, Türkova and Zdrazil proposed a general workflow for SLC transporters by using *in-silico* modelling combined with data science [79].

## 3 LIMITATIONS AND PITFALLS

The limitations of *in silico* approaches are important to consider before applying them. As shown before by various case studies, QSAR can be used for many tasks studying transporter proteins. However, it still has its limits in handling experimental errors regarding training and validating QSAR models [80]. The selection of relevant descriptors is key to avoiding intercorrelations of them, which negatively influences the final model [81]. Furthermore, it has to be taken into account that a model can only be used for test compounds that are in the chemical space of the model. Therefore, the applicability domain of the model has to be accessed. Aside from that, the accuracy of the prediction and the chance correlation have to be provided as well as a thorough external validation is necessary to make these models also applicable to regulatory applications [82].

Pharmacophore modeling is used amongst others in virtual screening. The disadvantage is that there are no good scoring metrics available to validate the results. In molecular docking, the affinity of a compound can be predicted using a scoring function, but in pharmacophore modeling only the root mean square deviation is used [83]. Furthermore, this approach depends on a database with only a restricted number of low-energy conformations per compound so someone cannot be sure that the active conformation is captured [84, 85] or that the provided data is accurate [86, 87]. Therefore, expert knowledge is needed and should be included as an additional step. Given that it improves the selection of targets as well as the assessment of an ADMET profile, it is still difficult to study new molecule classes since there is no information available for these compounds/targets [87-89].

Molecular docking is amongst others used in the pharma industry to find possible hit compounds in drug discovery. This method has benefited from the fact that the number of protein-ligand complexes gained from crystallography has increased. Therefore, the study of interaction patterns could be improved, and so key amino acids could be detected. To predict relevant binding modes of small compounds in the binding site, it is important to identify molecular interactions relevant to a high affinity. Furthermore, this can be used for the lead optimization stage to reduce the number of compounds that needs to be tested experimentally. The limits of this method are to find the best scoring function applicable for the used approach, to find suitable binding modes, and that the ranking of the compounds reflects the activity towards the target protein [90].

Molecular dynamics simulations give a deeper inside into the molecular nature of a protein-ligand complex by providing the interaction pattern and the strength of interactions over time. Furthermore, binding affinities can be determined with various methods, which are varying in their complexity and liability compared to experimental findings. This additional layer of complexity comes with an increase in simulation times as well as the need for more expensive hardware. Therefore, the size of the system that can be simulated is limited, meaning that large proteins, including more than one monomer, cannot be often simulated with all subunits. Instead, only the part of the protein, which is important for binding, is investigated in more detail [91].

## CONCLUSION

Transporters represent one of the last white spots in our target landscape. However, entering the era of big public data and simultaneously improving structural genomics technologies to allow the derivation of high-resolution structures of transmembrane proteins open the whole portfolio of ligand- and structure-based design methods. In public-private partnerships consortia, such as Resolute, the pharmaceutical industry, and academia join forces to deorphanize a large portion of the SLC transporter family. With cryogenic electron microscopy becoming almost a routine method, a lot of high-resolution structures of transmembrane transporter are expected to be released in the coming years. Also, the development of AlphaFold, an AI tool that generates three-dimensional structures based on only the amino acid sequence, can help to provide structures even when no similar structure is available. It offers a database with predicted protein structures based on physical and biological knowledge with high accuracy as well as a tool to generate customized models. [92]. Nevertheless, this approach has some limitations, such as no information about interactions with partner proteins being available as well as ligands, metal ions, and co-factors, which are important for their function and correct fold. Also, predictions of multimers show different accuracies [93]. These innovations will pave the way to uncover the molecular mechanisms of drug and nutrient transport across membranes and thus aid the design and development of new tool compounds and drugs. Furthermore, the role of a transporter in ADME and toxicity needs to be characterized in more detail, which will improve current toxicity prediction tools and thus reduce the number of animal experiments in the drug development process. First attempts in this direction, *i.e*., combining predicted BSEP inhibition profiles with classical machine learning to assess the risk of a compound causing cholestasis, showed encouraging results [94].

## Figures and Tables

**Fig. (1):**
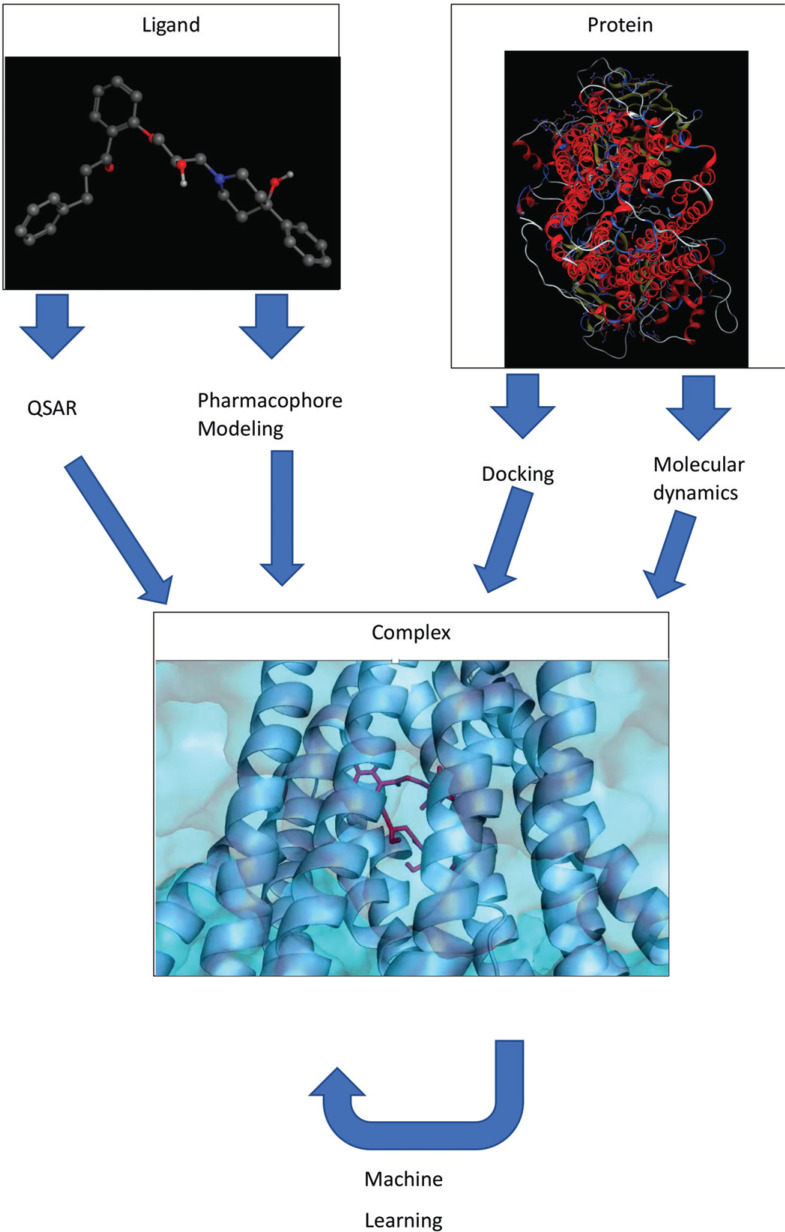
Overview of the *in silico* methods discussed in this review. Each method uses a different starting point, while QSAR and pharmacophore modeling approaches often start with a ligand structure. Docking and molecular dynamics simulations use the protein structure for their application. The output of these methods can be combined to obtain a deeper look into the interaction pattern of a protein-ligand complex. Additionally, approaches like Machine Learning can be used to support ligand- and structure-based methods.

**Fig. (2):**
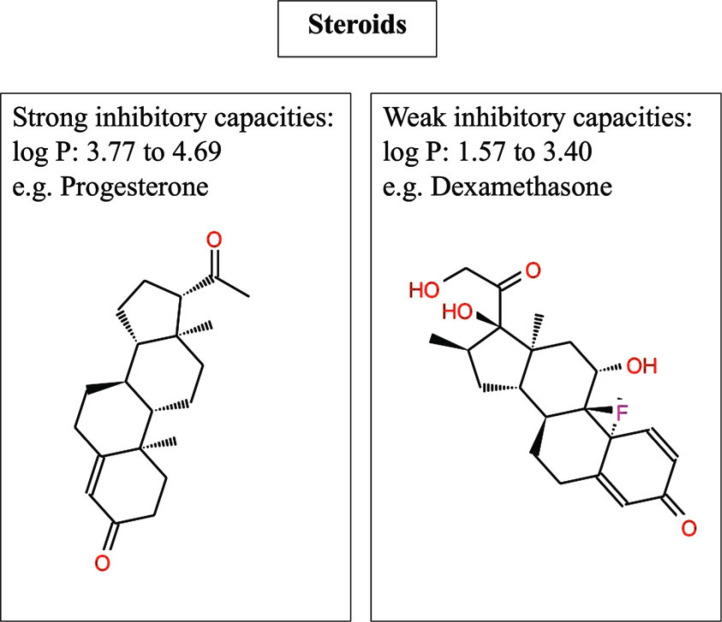
Steroids acting as inhibitors for P-gp. The study by Li *et al*. [19] revealed the difference between a strong and a weak inhibitor for P-gp. The difference in lipophilicity is the reason for the activity distinction. While compounds (*e.g*., Progesterone) with a higher logP (range between 3.77 to 4.69) show a stronger inhibition capacity, compounds like Dexamethasone with a lower value (range between 1.57 to 3.40) exhibit low inhibitory activity.

**Fig. (5):**
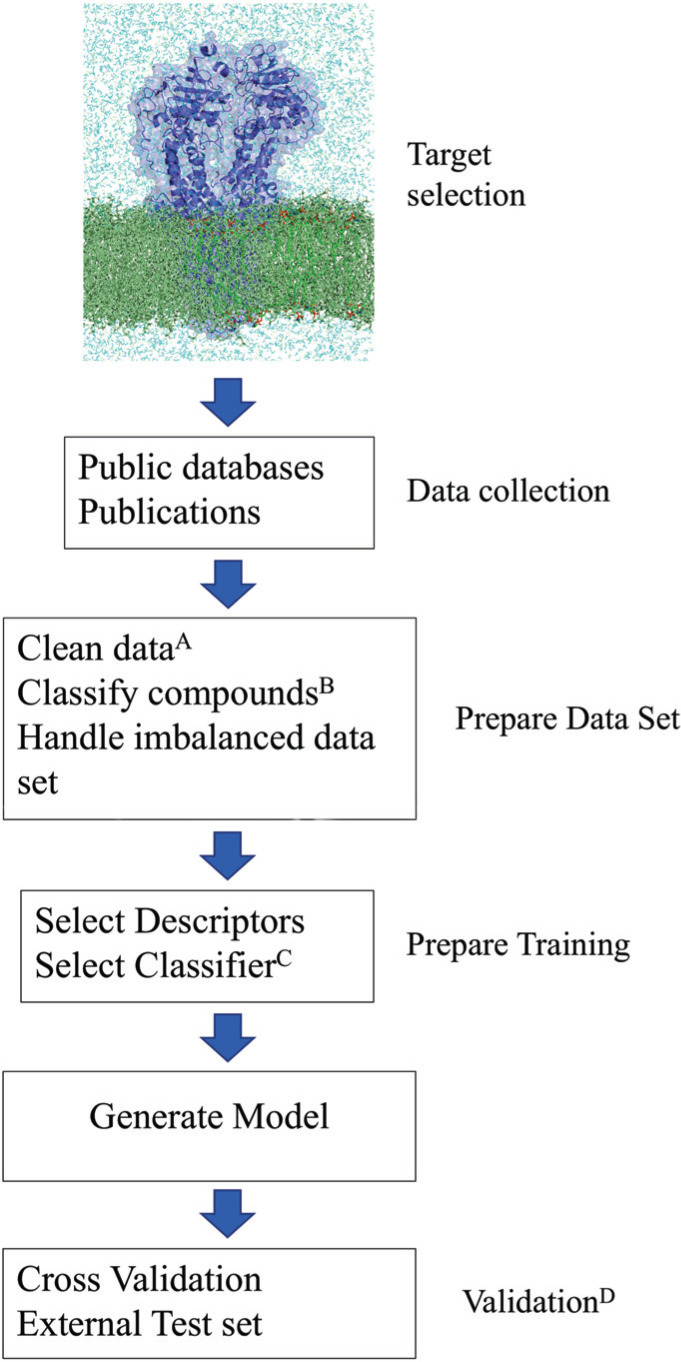
Building machine learning models. The first step to building a machine learning model is to identify the target of interest, which, for *e.g*., is involved in a certain disease or a key protein causing severe side effects. In the next step, data relevant to this target have to be collected (*e.g*. possible or known inhibitors or substrates for this protein). The collected data needs to be pre-processed to provide a data set with high quality. Before building a model, relevant descriptors and a classifier that represent the data need to be selected to train a model with high accuracy and broad chemical space. In the final step, the generated model has to be validated by performing a cross-validation and if available with an external test set. The scheme is partially derived from the steps used for building the machine learning models provided at www.livertox.univie.ac.at. On the website, detailed documentation is provided as an example for every step (*e.g*. used databases, publications, data set preparation, selected descriptors and classifiers, data set used for training). (**A**) Remove duplicates and compounds with inconsistent activity values. (**B**) Classify compounds in actives and in-actives depending on a transporter-specific threshold. (**C**) Classifiers should be selected specifically for each target protein. (**D**) For each target, cross-validation should be performed to validate the final model. If enough data is available, the model should also be validated with an external test set.

**Fig. (6):**
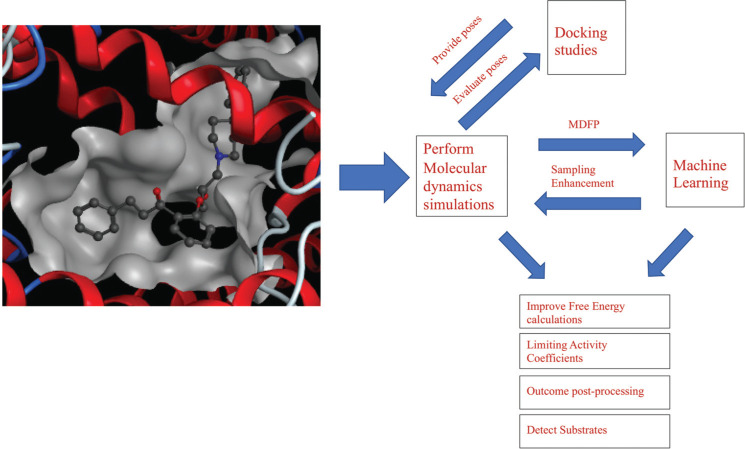
Combining molecular dynamics simulations with other *in silico* approaches. Molecular docking can provide various poses of protein-ligand complexes to enhance the number of starting structures for MD simulations. Furthermore, MD simulations can be used to validate the stability of certain docking poses revealed from docking by studying these interactions over time. The information obtained from simulations can be extracted to generate new descriptors to improve machine learning models. Also, machine learning can be used to enhance the sampling of MD simulations as well as make predictions to improve the parameterization of compounds.

**Table 1:** Transporters routinely tested in the drug development process.

**Transporters**	**Alias**	**Gene Name**	**Superfamily**
P-glycoprotein	P-gp	ABCB1	ATP-binding cassette transporter
Breast cancer resistance protein	BCRP	ABCG2	ATP-binding cassette transporter
Organic anion transporting polypeptide 1B1/1B3	OATP1B1/OATP1B3	SLCO1B1/SLCO1B3	Solute carrier
Organic anion transporter 1/3	OAT1/OAT3	SLC22A6/8	Solute carrier
Multidrug and toxin extrusion proteins	MATE	SLC47A	Solute carrier
Organic cation transporter 2	OCT2	SLC22A2	Solute carrier

**Note:** *[10].

## LIST OF ABBREVIATIONS

(Q)SAR: (Quantitative) Structure-activity Relationship
ABC transporter: ATP Binding Cassette Transporter
ADME-Tox: Absorption, Distribution, Metabolism, Excretion, Toxicity
ASP: Astex Statistical Potential
BCRP (ABCG2): Breast Cancer Resistance Protein
BGT: Betaine/Gamma-aminobutyric Acid Transporter
BSEP: Bile Salt Export Pump
DAT: Dopamine Transporter
ECFP: Extended Connectivity Fingerprint
GABA receptor: Gamma-aminobutyric Acid Receptor
GAT: Gamma-aminobutyric Acid Trans porter
GPU: Graphics Processing Unit
IMI: Innovative Medicines Initiative
KNIME: Konstanz Information Miner
MD: Molecular Dynamics
MDFP: Molecular Dynamics Fingerprints
MDR1: Multidrug Resistance Protein 1
Metrabase: Metabolism and Transport Database
ML: Machine Learning
MRP1: Multidrug Resistance-associated Protein 1
MRP2: Multidrug Resistance-associated Protein 2
OAT1 (SLC22A6): Organic Anion Transporter 1
OAT3 (SLC22A8): Organic Anion Transporter 3
OATP2B1: Organic Anion Transporting Polypeptide 2B1
OCT2 (SLC22A2): Organic Cation Transporter 2
Open PHACTS: Open Pharmacological Concepts Triple Store
P-gp(ABCB1): P-glycoprotein
PCM: Proteochemometrics Modeling
RESOLUTE: Research Empowerment on Solute Carriers
SERT: Serotonin Transporter
SLC: Solute Carrier Transporter
SVM: Support Vector Machine
TCDB: Transporter Classification Database
